# Prevalence and associated factors of mental disorders among prisoners in Mbarara municipality, southwestern Uganda: a cross-sectional study

**DOI:** 10.1186/s12888-019-2167-7

**Published:** 2019-06-11

**Authors:** Jimmy Ben Forry, Scholastic Ashaba, Godfrey Zari Rukundo

**Affiliations:** 1 Department of Psychiatry, Faculty of Medicine, Mbarara University of Science and Technology, P.O Box 1410, Mbarara, Southwestern Uganda Uganda; 2 Department of Mental Health and Psychiatry, Faculty of Medicine, Kampala International University, P.O Box 71, Bushenyi, Southwestern Uganda Uganda; 3 Mubende Regional Referral Hospital, P.O Box 4, Mubende, Central Uganda Uganda

**Keywords:** Crime, Mental disorders, Mental health, Prisoners, Uganda

## Abstract

**Background:**

Research in high income countries reports higher prevalence of mental disorders among prisoners than in the general population. Lack of published data from low resource settings affects planning and eventual service provision to the prisoners. This study aimed to determine the burden of mental disorders and associated factors among prisoners in Mbarara municipality in southwestern Uganda.

**Methods:**

This was a prison facility based cross sectional study among 414 inmates in Mbarara municipality. We consecutively enrolled them by simple random sampling from 3 prison facilities. Participants completed a sociodemographic and clinical factor questionnaire, and the M.I.N.I. Version 6.0. Data were analyzed using STATA 12.0. Univariate, bivariate and multivariate logistic regression analyses were conducted to determine the prevalence, and associated factors using the individual odds ratios with their 95% Confidence Intervals and *P*-values as a measure of association, clinical and statistical significance respectively.

**Results:**

A total of 354 (86%) prison-inmates met criteria for a psychiatric disorder. Of these, 338 (95%) had one or more diagnoses. Major depression was the most common diagnosis (44%). Factors associated with mental disorders included history of traumatic brain injury (OR = 2.57; 95% CI = 1.22–5.42; *P*-value = 0.01), low income status (OR = 0.32; 95% CI = 0.16–0.63; *P*-value = 0.001) and authoritarian parenting (OR = 0.37; 95% CI = 0.18–0.75; *P*-value = 0.006).

**Conclusions:**

There is a high prevalence of psychiatric illness among prisoners in Mbarara municipality with most of them having more than one diagnosis. Majority of the prisoners with mental illness go undiagnosed and untreated.

## Background

There is a paucity of data about mental disorders among prisoners in low resource settings despite the existence of high numbers of prisoners [1–3]. Data from high income countries report a high prevalence of mental disorders among prisoners with developing countries carrying the biggest burden [4]. The prevalence of psychiatric disorders is greater among prisoners when compared to the general population [1, 4–6]. The mental health needs of prisoners should be paramount regardless of whether the inmate developed these psychiatric symptoms while in prison or prior to the incarceration [7].

Most common factors associated with mental disorders among prisoners include: a prior history of traumatic brain injury [8], recidivism and prior incarceration [9], being male [10], young and of a low level of education [11], being married [12] and alleged to have committed a violent crime [10, 13]. Since the factors listed above vary across different settings, findings in one setting may not apply to other settings. There is therefore need to conduct studies in various settings.

In Uganda, a low income country [14], the government owns and administers all prison facilities. The prisons have health facilities that provide services targeting general medical conditions and have no psychiatric services. Psychiatric cases and assessments are referred to nearby regional referral hospitals that offer mental health services. Butabiika National Referral Hospital is the only mental health hospital in the country and has a forensic inpatient unit having a bed capacity of 116 beds with only about 10% of the beds in this unit occupied by the institutionalized mentally-ill prisoners, some for more than 5 years [15]. The national incarceration rate for 2014 was 121 per 100,000 persons and has been on the rise since 2010 [16]*.* This was greater than the average incarceration rates for Africa and Asia, and slightly below the average global incarceration rate of 144 per 100,000 of the national populations [3]. When compared to other African countries in terms of top rankings for national incarceration rates, Uganda was in the 18th position sharing it with Cameroon [3]. Health screening of prisoners is often conducted at the time of admission into the various prison facilities focusing on general medical conditions without regard for psychiatric disorders. This is probably due to poorly equipped health facilities with an absence of trained mental health care providers [17]. Other factors that result in the incarceration of the mentally ill include deinstitutionalization, stringent judicial practices, inadequate community support, mentally ill offenders’ limited access to community treatment, and the attitudes of police officers and society as a whole [18] despite the enshrinement of the McNaughten rule and basic human rights in Uganda’s constitution [19].

Uganda has a high number of prisoners, with the southwestern region having one of the highest incarceration rates in the country [3, 16]. However, there is a lack of published studies that have been conducted among Ugandan prisoners nationwide or in the region. Therefore this study aimed to determine the burden of mental disorders and associated factors among prisoners in Mbarara municipality, southwestern Uganda.

## Methods

### Aim

The study sought to determine the prevalence of mental disorders among prisoners in Mbarara municipality, southwestern Uganda and identify the associated factors.

### Design

This was a prison facility based study with a cross sectional research study design.

### Setting

Southwestern Uganda has several districts with Mbarara being one of the most densely populated. Mbarara municipality, which is the main third order administrative division, is 1445 m above sea level, found along the Kampala-Kabale highway and is 266 km from Kampala, Uganda’s capital and largest city. The municipality consists of 6 divisions that include Biharwe, Kakiika, Kakoba, Kamukuzi, Nyakayojo and Nyamitanga. Mbarara Central and Mbarara Women Prisons are situated in Kiswahili cell, Nyamitanga division in Mbarara municipality, about 100 m from the Mbarara-Kabale highway while Kyamugorani Prison is located in Kyamugorani, Kakiika division, Mbarara municipality.

### Characteristics of study participants

Prison inmates in Mbarara municipality are incarcerated in two male and one female government funded and administered prisons called Kakiika, Mbarara Main and Mbarara Women Prisons respectively. Mbarara Main Prison is one of the largest prison facility in the region with a population of 1637, followed by Kakiika Prison with 742 and then Mbarara women Prison with 165 occupants. Of these, 70% were on remand, 23% were convicted but not sentenced and 7% were sentenced. The inmates considered for possible recruitment into the study were aged eighteen years and above regardless of their prison status, with no hearing or speech impediments. The refusal rate for participation in the study was 0.7% (2 males and 1 female) and of the potential participants excluded from the study; 19 males were absent, 3 males were below 18 years of age, 2 males had speech impairments and 1 male had a hearing impairment. There weren’t participants who could not be included in the study due to acute mental illness as they were either too ill to be interviewed or could not give informed consent.

### Measurement of mental disorders among prisoners

We conducted a descriptive cross-sectional study among prisoners in three prison facilities in Mbarara municipality, southwestern Uganda from June to July, 2017. We recruited 8 research assistants who were mostly social workers with the exception of 2 i.e. a psychiatric clinical officer and a psychiatric nurse. They undertook the entire process under the supervision and guidance of the principal investigator after undergoing a weeklong daily didactic and practical training sessions on the use and administration of the study tools including the M.I.N.I Version 6.0. The principal investigator, who was a psychiatry resident at the time of data collection, was readily available and directly involved in the research process and verification of the responses. He was responsible for the formulation of the individual current diagnoses.^1^ The study participants were interviewed once in a clinical setting with privacy and confidentiality in mind whilst adhering to the standard safety precautions, prison regulations and code of conduct. The sociodemographic, forensic and clinical factors of the study participants such as parenting style,^2^ past traumatic brain injury,^3^ category of crime,^4^ past psychological trauma^5^ and available follow-up services^6^ were obtained by a specially designed interviewer administered questionnaire. A diagnosis of a mental disorder was attained by participants responding to queries in the MINI Version 6.0 and appropriate conclusions and diagnoses arrived at by the principal investigator based on their various responses without a need for an additional clinical interview.

### Sampling design

The sample size was determined by the Kish Leslie formula [20] using a prevalence of 55.4% reported in a study conducted in South Africa [21]. The minimum sample size was 380 respondents. In order to account for incompleteness or loss of information, we increased the number by 9% to have a total of 414 respondents. The study participants were selected according to the population proportions of the three prisons in the ratio of 10:4:1 for Mbarara Main, Kakiika and Mbarara Women Prisons respectively. Participant recruitment was by simple random sampling. The individual prison registries were used focusing on inmates present at the time of data collection. Every fifth inmate was chosen until the required number was attained. The respondents were recruited after obtaining informed consent from each one of them. The questionnaire and MINI Version 6.0 were administered in private and confidentially.

### Statistical analysis

The data collected was checked for completeness, coded and entered into Epidata manager version 2.0.8.56 and then transferred to STATA 12.0 for univariate, bivariate and multivariate analyses. Univariate analyses were done for the socio-demographic and clinical factors as well as to estimate the prevalence. Bivariate and multivariate logistic regression analyses were conducted to determine associations between the category of mental illness, and the associated factors using the individual odds ratios with their 95% Confidence Intervals and *p*-values. The results were considered statistically significant if the *p*-value was less than 0.05 and clinically significant if the 95% Confidence Interval was a narrow range and did not cross the line of no difference.

## Results

### Description of respondents

Majority of the 414 respondents in this study were male (94%), aged 22–35 years (60%), married or cohabiting (53%), with a primary school education (67%) and belonging to the low income class (72%). Most of them were first-time offenders (89%) and had no access to legal representation (82%). Majority were incarcerated under regular imprisonment^7^ (55%), with more than half (53%) undergoing health screening for physical ailments at the time of admission into their various prison facilities, while 64% were alleged to have committed or were convicted of violent crimes. Many of them (88%) had sought health care services from the prison health facilities with 7% having ever sought professional psychiatric assistance, 3% currently seeking psychiatric treatment and another 3% having ever sought other forms of treatment for their psychiatric illnesses.

### Prevalence of mental disorders among prisoners

Only 13% (*n* = 53) had a single mental disorder (current,^8^ past^9^ and lifetime^10^) while 73% (*n* = 301) having more than one diagnosis of a mental disorder (current, past and lifetime). The overall lifetime prevalence of mental disorders was 86% (*n* = 354) with 95% (*n* = 338) of these having one or more current episodes. Of these, major depression was the most common individual current diagnosis (44%), followed by post-traumatic stress disorder (31%), suicidality^11^ (25%), psychotic disorder (22%) and antisocial personality disorder (21%) (Table 1).

**Table 1:** Prevalence of individual current diagnoses and comorbid mental disorders among prisoners in Mbarara municipality, July 2017 (*N* = 414)

ICD10 F Categories	Variable	n (%)
	Major depressive disorder	183 (44.2)
F32.x	Current (2 weeks)	36 (8.7)
F32.x	Past	53 (12.8)
F33.x	Recurrent	94 (23)
	Bipolar affective disorder I	40 (9.7)
F30.x – F31.9	Current	12 (2.9)
F30.x – F31.9	Past	28 (6.8)
	Bipolar affective disorder II	57 (13.8)
F31.8	Current	26 (6.3)
F31.8	Past	31 (7.5)
	^a^Mood disorder with psychotic features	40 (9.7)
F32.3/F33.3/F30.2/F31.2/F31.5	Lifetime	11 (2.7)
F31.8/F31.9/F39	Current	29 (7.0)
	Psychotic disorder	92 (22.2)
F20.xx – F29	Lifetime	24 (5.8)
-	Current	68 (16.4)
	Suicidality Current (Past Month)	102 (24.6)
-	Low	43 (10.4)
-	Moderate	15 (3.6)
-	High	44 (10.6)
F40.00	Agoraphobia (Current)	55 (13.3)
F41.1	Generalized anxiety disorder Current (Past 6 Months)	128 (30.9)
F42.8	Obsessive compulsive disorder Current (Past Month)	53 (12.8)
F43.1	Post-traumatic stress disorder Current (Past Month)	128 (31)
	Panic disorder	135 (32.6)
F40.01 – F41.0	Current (Past Month)	82 (19.8)
-	Lifetime	53 (12.8)
	Social phobia/Social anxiety disorder Current (Past Month)	80 (19.3)
F40.1	Generalized	58 (14.0)
F40.1	Non-generalized	22 (5.3)
	Substance use disorders	52 (12.5)
	Alcohol use disorder (Past 12 Months)	45 (10.8)
F10.2x	Dependence	42 (10.1)
F10.1	Abuse	3 (0.7)
	Non-alcohol use disorder (Past 12 Months)	7 (1.7)
F11.1 – F19.1	Dependence	5 (1.2)
F11.1 – F19.1	Abuse	2 (0.5)
-	Psychiatric disorders due to a general medical condition (Organic/Drug-related)	5 (1.2)
	Uncertain	1 (0.2)
F60.2	Antisocial personality disorder (Lifetime)	85 (20.5)
-	Major depression with post-traumatic stress disorder	88 (21)
-	Major depression with suicidality	61 (15)
-	Psychotic disorders with suicidality	32 (8)

^a^Mood disorder with psychotic features refers to a study participant having symptoms of a primary psychiatric disorder at the time of administering and as determined by the responses to the M.I.N.I Version 6.0 that meet the criterion for a psychotic disorder as well as a mood disorder

### Factors associated with mental disorders among prisoners

After multiple logistic regression (Table 2), a history of past traumatic brain injury (Adjusted Odds Ratio = 0.299; 95% CI = 0.106–0.843; *P*-value = 0.022) and being convicted but not sentenced (Adjusted Odds Ratio = 0.22; 95% CI = 0.05–0.93; P-value = 0.04) were the only factors that were statistically significant with regards to a single diagnosis of mental illness amongst prison-inmates incarcerated in Mbarara municipality.

**Table 2:** Factors associated with a single diagnosis of mental illness among prisoners in Mbarara municipality, July 2017 (*N* = 414)

Variable	n (%)	Unadjusted Odds Ratio (95% CI)	*P*-value	Adjusted Odds Ratio (95% CI)	*P*-value
Past traumatic brain injury					
Yes	119 (28.7)	0.29 (0.12–0.69)	0.005*	0.32 (0.10–0.96)	0.04*
No	295 (71.3)	1.00 -	–	1.00 -	–
Parenting style					
Authoritarian	143 (34.5)	1.69 (0.77–3.73)	0.19	2.00 (0.82–4.93)	0.13
Authoritative	119 (28.7)	0.52 (0.19–1.44)	0.21	0.77 (0.25–2.36)	0.65
Permissive	58 (14.0)	1.96 (0.78–4.97)	0.15	1.92 (0.69–5.39)	0.21
Neglectful/Uninvolved	94 (22.7)	1.00 -	–	1.00 -	–
Available follow-up services					
Outreach	22 (5.3)	5.06 (0.91–28.15)	0.06	3.39 (0.46–24.83)	0.23
Prison facility	363 (87.7)	1.86 (0.43–8.10)	0.41	1.06 (0.19–5.96)	0.95
Mbarara Regional Referral Hospital	29 (7.0)	1.00 -	–	1.00 -	–
Prison status					
On remand	292 (70.5)	0.75 (0.27–2.09)	0.58	0.46 (0.14–1.54)	0.21
Convicted not sentenced	94 (22.7)	0.31 (0.09–1.12)	0.07	0.22 (0.05–0.93)	0.04*
Sentenced	28 (6.8)	1.00 -	–	1.00 -	–
Category of alleged/confirmed crime					
Violent	265 (64.0)	0.51 (0.10–2.58)	0.42	0.76 (0.11–5.08)	0.78
Non-violent	140 (33.8)	0.45 (0.09–2.36)	0.35	0.61 (0.09–4.18)	0.61
Drug-related	9 (2.2)	1.00 -	–	1.00 -	–
Past psychological trauma					
Yes	190 (45.9)	0.64 (0.35–1.17)	0.150	0.82 (0.36–1.84)	0.63
No	224 (54.1)	1.00 -	–	1.00 -	–

* Statistically significant at *P*-value < 0.05

A similar multiple logistic regression model (Table 3) was utilized for all dependent variables to determine factors that influence the presence of more than one mental disorder among prisoners in Mbarara municipality and it was found that a total of five factors were statistically significant: having a low income status (OR = 0.32; 95% CI = 0.16–0.63; *P*-value = 0.001), past traumatic brain injury (OR = 2.57; 95% CI = 1.22–5.42; P-value = 0.01), solitary confinement (OR = 0.35; 95% CI = 0.16–0.74; P-value = 0.006), and being raised by authoritarian parents/guardians (OR = 0.37; 95% CI = 0.18–0.75; *P*-value = 0.006).

**Table 3:** Factors associated with more than one diagnosis among the study participants

Variable	n (%)	Unadjusted Odds Ratio (95% CI)	*P*-value	Adjusted Odds Ratio (95% CI)	*P*-value
Past traumatic brain injury	119 (28.7)				
Yes	295 (71.3)	2.89 (1.64–5.11)	0.000*	2.57 (1.22–5.42)	0.01*
No		1.00 -	–	1.00 -	–
Parenting style	143 (34.5)				
Authoritarian	119 (28.7)	0.57 (0.31–1.03)	0.06	0.37 (0.18–0.75)	0.006*
Authoritative	58 (14.0)	1.14 (0.59–2.20)	0.70	0.66 (0.29–1.48)	0.31
Permissive	94 (22.7)	0.55 (0.26–1.13)	0.10	0.50 (0.21–1.18)	0.11
Neglectful/Uninvolved	119 (28.7)	1.00 -	–	1.00 -	–
Socioeconomic status (Annual income)					
Low-income (< 1 million UgShs)	298 (72.0)	0.47 (0.27–0.80)	0.006*	0.32 (0.16–0.63)	0.001*
Middle-income (1–5 million UgShs)	114 (27.5)	1.00 -	–	1.00 -	–
Upper-income (> 5 million UgShs)	2 (0.5)	1.00 -	–	1.00 -	–
Prison status					
On remand	292 (70.5)	1.77 (0.80–3.89)	0.16	2.07 (0.83–5.12)	0.12
Convicted not sentenced	94 (22.7)	4.28 (1.67–10.96)	0.002*	4.87 (1.66–14.26)	0.004**
Sentenced	28 (6.8)	1.00 -	–	1.00 -	–
Current living situation in prison					
Regular incarceration	228 (55.1)	0.91 (0.54–1.54)	0.72	0.68 (0.34–1.34)	0.27
Solitary confinement	70 (16.9)	0.43 (0.22–0.81)	0.01*	0.35 (0.16–0.74)	0.006*
Incarceration with hard labour	116 (28.0)	1.00 -	–	1.00 -	–

*Statistically significant at *P*-value < 0.05

**Confounding noted

Socioeconomic status refers to the mean monthly income as reported by a study participant in the year prior to their current incarceration

## Discussion

This study assessed the prevalence of psychiatric morbidity and associated factors among prisoners in Mbarara municipality, southwestern Uganda. The prevalence of a single diagnosis was 13% whereas the prevalence of more than one diagnosis was 73%. We also found a prevalence of 95% for one or more current episodes and 86% for lifetime, past and current episodes of mental disorders. Overall major depressive disorders (44%), post-traumatic stress disorders (31%) and antisocial personality disorders (21%) were the most common individual mental disorders diagnosed.

The prevalence of mental disorders in this study of 86% is quite high compared to that found in the general population of 30% [5] but similar to what has been reported in previous prison studies with similar trends in individual current diagnoses [1, 12, 22, 23]*.* A high rate of suicidality is also consistent with the published prison mental health literature [8, 24]. Suicidality was also demonstrated to occur more often in major depression than in psychotic disorders, a finding also in line with current literature [25]*.* And among the least diagnosed mental illnesses were psychiatric disorders due to a general medical condition (1.2%), bipolar affective disorders type I (9.7%) and substance use disorders (12.5%). Previous studies in prison populations have reported a high prevalence of alcohol and other substance use disorders in Uganda as well as other countries [9, 26, 27]*.* This difference might be attributed to the rigorous security checks that visitors are subjected to and the formidable security measures in place that limit access to such substances of abuse presently in Ugandan prisons. The other possible explanation for the difference could be the need to avoid the repercussions of being reported to use substances of abuse, and the desire to reform and deal with the guilty conscience among prisoners who actually committed the crimes that they are accused of.

Majority of the individuals with mental illness were young [11, 12, 28] and first time offenders [13]*.* They had a low education level [8, 11, 12], were alleged or convicted of committing violent crimes [29] and had a past history of traumatic brain injury [8]. Previous studies report similar findings but with some exceptions such as substance use [30], a prior history of mental illness and a history of past psychological trauma such as child abuse [26]. The possible explanation for the observed discrepancy could be due to the stigma and discrimination associated with substance use, mental illness and child abuse as well as the fact that Uganda is a low income country [14] with markedly different sociodemographic and economic characteristics while the vast majority of the findings from studies reviewed have been conducted in middle and upper income countries [1]*.* In addition, inadequacies in the judicial systems due to a variety of factors [31]*,* as well as the inadequately equipped and overburdened health care systems [17] may also play a role.

The factors that were associated with mental illness in prisoners were low income status, incarceration under solitary confinement, past traumatic brain injury [8] and being raised by authoritarian parents or guardians. Prisoners with more than one diagnosis were more likely to have suffered a traumatic brain injury in the past [8] and to have been convicted but not sentenced, whereas inmates who were in solitary confinement, of a low income status and had been raised by authoritarian parents/guardians less likely to be diagnosed with two or more mental disorders.

The presence of a past traumatic brain injury was less likely among those diagnosed with a single mental disorder since fewer inmates had a single diagnosis. Traumatic brain injury is a known risk factor for mental illness and as such we expect it to be more likely in the majority i.e. those with more than one diagnosis. Most of the results from studies that were reviewed did not concur with the findings in this study and it can be postulated that the reason for this is the stark contrast attributed to differences in terms of study tools/instruments used, socioeconomic status, culture and judicial systems between Uganda and the other countries in which those studies were conducted.

The limitations encountered during the course of conducting this study include the fact that study participants comprised of only respondents incarcerated in the prison facilities in Mbarara municipality and so the findings cannot be generalized to other prisons. However given the fact that the living conditions and judicial system is similar, these findings would provide a basic insight into the nature and extent of the burden of mental illnesses in Ugandan prisons. Some respondents may have deliberately declined to disclose and/or falsified responses to some inquiries that they considered to be private, intimate, confidential and/or sensitive.

This could have been due to fear of reprisals and consequences by the prison authorities, anticipated exploitation of the sick role and the societal status and the ascribed privileges that accompanied it. This was mitigated by proper consenting of the study participants, sensitization of the non-professional psychiatric personnel, psychoeducation of the study participants, soliciting of psychiatric drugs and funds to provide mental health services in the correctional institutions. Any difficulties in obtaining accurate information about details in the past and long term symptoms were attributed to recall bias. Some inmates might have been malingering in order to assume the much coveted sick role and all its perceived benefits.

The lack of resources to confirm diagnoses of general medical conditions and ruling out psychiatric symptoms due to physical illnesses. Absence of some prisoners at the time of data collection due to prison scheduling e.g. community service, prison duties, court schedules and pending releases from prison may have also impacted the results. Aspiring to attain the mandated sample size while endeavoring to cater for potential data loss with the intension of getting a study population that is representative of the general prison population that I was planning to study went a long way to resolve these concerns.

## Conclusions

There is a high prevalence of psychiatric illnesses among prisoners in Mbarara municipality with most of them having more than one diagnosis. Most of the prisoners with mental illnesses go undiagnosed and untreated. A past history of traumatic brain injury is a risk factor for having more than one diagnosis of a psychiatric disorder.

The findings of this study indicate that there is a need for capacity building for health workers and other staff in prisons in regard to screening, assessing and treatment of inmates with mental disorders. In addition, there is a necessity for a clear referral process for individuals found to have mental disorders*.* Furthermore, improving the conditions and standards of living for those incarcerated individuals would help in preventing mental disorders and their comorbidities. Lastly, the field of forensic psychiatry in Uganda would significantly benefit from more comprehensive, longitudinal and nationwide studies to attain a clear picture of the actual magnitude of the burden of mental disorders in Ugandan prisons.

## Data Availability

The dataset used and analyzed during the current study is available from the corresponding author on reasonable request.

## Abbreviation

DSM-IV-TR: Diagnostic and Statistical Manual of Mental Disorders Fourth Edition Text Revised
ICD-10: International Statistical Classification of Diseases and Related Health Problems Tenth Revision
MINI: Mini International Neuropsychiatric Interview

## Footnotes

1 Individual current diagnosis refers to a major primary psychiatric disorder diagnosed in a study participant at the time of administering and as determined by the responses to the M.I.N.I Version 6.0 regardless of the timing, duration, frequency, comorbidity or presence of other multiple diagnoses.

2 Parenting style refers to the type of upbringing that the participant underwent and this was assessed by asking the study participant to briefly describe the nature of interaction with their parents or guardians.

3 Past traumatic brain injury refers to a study participant reporting a history of head trauma associated with either loss of consciousness, nausea and/or vomiting, open wound, raccoon eyes, bleeding or discharge from the ears and/or nose.

4 Category of crime refers to the classification of the offense of which the study participant is alleged to have committed or is convicted of, as either violent, non-violent or drug-related, and this was assessed by asking the participant the nature of the offense of which he/she was accused.

5 Past psychological trauma refers to a study participant reporting a history of subjective or clinically significant distress that impaired their social or occupational functioning following witnessing or involvement in a physically or emotionally traumatic event but does not fulfill the DSM-IV-TR criteria for any mental disorder.

6 Available follow-up services refer to general and mental health services that are readily available and have been accessed by the study participants for follow-ups during incarceration as reported by the participant, and where they are found with options including prison-based outreach programs by non-prison health workers, the nearest regional referral hospital i.e. Mbarara, and the prison health facility.

7 Regular incarceration refers to the current living situation in prison as reported by the study participants that does not include solitary confinement or incarceration with hard labor.

8 Current episode refers to a study participant having psychiatric symptoms as determined by the responses to the queries in the M.I.N.I Version 6.0 and present at the time of administering the M.I.N.I that meet the diagnostic criterion for a particular psychiatric disorder as per DSM-IV-TR.

9 Past episode refers to a study participant having a history of psychiatric symptoms as determined by the responses to the queries in the M.I.N.I Version 6.0 and at the time of administering the M.I.N.I that meet the diagnostic criterion for a particular psychiatric disorder as per DSM-IV-TR.

10 Lifetime episode refers to a study participant having psychiatric symptoms at least once in their lifetime as determined by the responses to the queries in the M.I.N.I Version 6.0 and at the time of administering the M.I.N.I that meet the diagnostic criterion for a particular psychiatric disorder as per DSM-IV-TR.

11 Suicidality refers to the presence and severity of suicidal ideations, suicidal intent with or without an associated suicidal plan as well as suicidal attempts in a study participant within the past 1 month as determined by the responses to the queries in the M.I.N.I Version 6.0.

## References

[1] Fazel S, Seewald K (2012). Severe mental illness in 33 588 prisoners worldwide: systematic review and meta-regression analysis. Br J Psychiatry.

[2] Walmsley R (2009). World prison population list, 8th edn.

[3] Walmsley R., World prison population list 2015, International Centre for Prison Studies/University of Essex, Recuperado el: Birkbeck University of London.

[4] Davis, M.R., et al., The identification of mental disorders in the criminal justice system. 2007: Australian Institute of Criminology.

[5] Kasoro S (2002). Mental illness in one district of Uganda. Int J Soc Psychiatry.

[6] Statistics, A.B., National Survey of Mental Health and Wellbeing: Summary of Results, 2007. 2008, Canberra.

[7] Salize, H.J., H. Dreßing, and C. Kief, Mentally disordered persons in European prison systems–Needs, programmes and outcome (EUPRIS). Final report. Mannheim, central institute of Mental Health, 2007.

[8] Sepehrmanesh Z, et al. Prevalence of psychiatric disorders and related factors in male prisoners. Iran Red Crescent Med J. 2014;16(1). 10.5812/ircmj.15205 PMC3964429 24719711

[9] El-Gilany A-H (2016). Psychiatric disorders among prisoners: a national study in Egypt. East Asian Arch Psychiatr.

[10] Baillargeon J (2009). Psychiatric disorders and repeat incarcerations: the revolving prison door. Am J Psychiatr.

[11] López M, et al. Prevalence of mental health problems in sentenced men in prisons from Andalucía (Spain). Rev esp sanid penit. 2016:76–84. 10.4321/S1575-06202016000300002 27831595

[12] Zabala-Baños M (2016). Mental disorder prevalence and associated risk factors in three prisons of Spain. Revista espanola de sanidad penitenciaria.

[13] Ibrahim EM (2014). Psychiatric morbidity among prisoners in Egypt. World J Med Sci.

[14] Fantom, N. and U. Serajuddin, The World Bank’s classification of countries by income. 2016.

[15] Kigozi F (2010). An overview of Uganda's mental health care system: results from an assessment using the world health organization's assessment instrument for mental health systems (WHO-AIMS). Int J Ment Heal Syst.

[16] UBS (2015). Statistical Abstract, U.B.o. Statistics, Editor. 2015.

[17] Todrys, K.W. and S.-R. Kwon, " Even dead bodies must work": health, hard labor, and abuse in Ugandan prisons. 2011: Human Rights Watch.

[18] Erickson CD (2012). Using systems of care to reduce incarceration of youth with serious mental illness. Am J Community Psychol.

[19] The Prisons Act, 2006, in 2012 No. 65. 2006, UPPC, Entebbe: Uganda.

[20] Kish, L., Survey sampling. 1965.

[21] Naidoo S, Mkize D (2012). Prevalence of mental disorders in a prison population in Durban, South Africa. Afr J Psych.

[22] Gonçalves LC (2016). A longitudinal study of mental health symptoms in young prisoners: exploring the influence of personal factors and the correctional climate. BMC Psychiatry.

[23] Aishatu, Y., et al., Prevalence of psychiatric morbidity among inmates in Jos maximum security prison. 2013.

[24] Goss JR, et al. Characteristics of suicide attempts in a large urban jail system with an established suicide prevention program. Psychiatr Serv. 2002. 10.1176/appi.ps.53.5.574 11986506

[25] Ayhan G (2017). Suicide risk among prisoners in French Guiana: prevalence and predictive factors. BMC Psychiatry.

[26] Marín-Basallote N, Navarro-Repiso C (2012). Study of the prevalence of severe mental disorder in the penitentiaries Puerto I, II and III of Puerto de Santa María (Cádiz): new strategies of psychiatric care in prison. Revista espanola de sanidad penitenciaria.

[27] Fazel S (2016). Mental health of prisoners: prevalence, adverse outcomes, and interventions. Lancet Psychiatry.

[28] Dadi AF (2016). Anxiety and associated factors among prisoners in north west of Amhara regional state, Ethiopia. BMC Psychiatry.

[29] Baillargeon J (2010). Risk of reincarceration among prisoners with co-occurring severe mental illness and substance use disorders. Adm Policy Ment Health Ment Health Serv Res.

[30] Eytan A, et al. Psychiatric symptoms, psychological distress and somatic comorbidity among remand prisoners in Switzerland. Int J Law Psychiatry. 2010:1–7. 10.1016/j.ijlp.2010.11.003 21126766

[31] USDS, Bureau of Democracy (2013). Uganda 2013 Human Rights Report, in Country Reports on Human Rights Practices for 2013, H.R.a.L.

